# Supplementary material for: Experimental quality control induces changes in Allen mouse brain connectomes
Source: Imaging Neurosci (Camb). 2026 Jul 28;4:IMAG.a.1310. doi: 10.1162/IMAG.a.1310 (PMC13417618; doi:10.1162/IMAG.a.1310)
Supplement: Supplementary Material [file IMAG.a.1310_supp.pdf]

Supplementary Data: Our rebuilt connectomes and QC images are publicly available at the following Zenodo repository: https://zenodo.org/records/20276701.

Data and Code Availability

All code is stored on https://github.com/vik16nathan/allen_connectome_qc. All input data is publicly available and downloaded from the Allen API. Our rebuilt connectomes are publicly available at the following Zenodo repository: https://zenodo.org/records/18745615.

Supplementary Tables

Supplementary Table 1: List of abbreviations for major division names.

**Full Major Division Name**	**Major Division Abbreviation**
Isocortex	Isocortex
Olfactory areas	OLF
Hippocampal formation	HPF
Cortical sublate	CTXsp
Striatum	STR
Pallidum	PAL
Thalamus	Thal
Hypothalamus	Hypothal
Midbrain	Midbrain
Pons	Pons
Medulla	Medulla
Cerebellum	CB

Supplementary Table 2: The number of experiments with injections in each major brain division before versus after QC.

Division	Before QC	After QC	Lost
Isocortex	128	121	7
OLF	21	19	2
HPF	49	39	10
CTXsp	8	7	1
STR	27	23	4
PAL	10	9	1
Thal	44	38	6
Hypothal	34	25	9
Midbrain	42	33	9
Pons	16	16	0
Medulla	39	35	4
CB	19	16	3

Supplementary Table 3: We replicate Table 1 from Knox et al., which calculates MSE_{rel} values, defined as “the mean square error relative to the average squared norm of the prediction and left-out data.” These values can range from 0-200% (Knox et al., 2018). In parentheses, we also show the change in error after QC. Bolded values indicate reductions in error after QC versus before QC.

Major division	Model	Voxel MSErel	Voxel MSErel, Training	Region MSErel	Region MSErel, Training	Region PTP	Region PTP, Training
Isocortex	Voxel	**63% (-3.4)**	12% (-10.0)	**33% (-1.0)**	7% (-4.8)	32% (0.6)	8% (-0.9)
Isocortex	Homogeneous			37% (0.3)	20% (-0.2)	**32% (-0.9)**	17% (-0.1)
OLF	Voxel	**74% (-7.8)**	15% (-1.6)	**40% (-1.0)**	7% (0.3)	43% (2.6)	6% (0.3)
OLF	Homogeneous			**42% (-7.5)**	8% (-0.8)	55% (10.7)	9% (-0.8)
HPF	Voxel	**80% (-12.0)**	30% (9.4)	**53% (-3.5)**	24% (6.9)	**52% (-0.6)**	23% (6.5)
HPF	Homogeneous			**44% (-1.6)**	48% (-3.4)	**42% (-6.2)**	48% (-2.9)
CTXsp	Voxel	**100% (-14.1)**	53% (12.9)	101% (6.4)	64% (17.3)	102% (9.5)	37% (11.6)
CTXsp	Homogeneous			**94% (-17.2)**	1% (-1.2)	**76% (-22.2)**	1% (-1.3)
STR	Voxel	110% (6.2)	3% (-4.7)	51% (5.7)	1% (-0.8)	44% (3.9)	0% (-0.6)
STR	Homogeneous			65% (12.1)	23% (-0.3)	65% (11.6)	23% (-1.3)
PAL	Voxel	109% (8.7)	8% (2.6)	74% (10.2)	4% (1.5)	45% (0.0)	7% (0.0)
PAL	Homogeneous			112% (27.2)	3% (-0.2)	74% (0.0)	2% (0.0)

TH	Voxel	117% (2.2)	8% (-6.3)	81% (4.2)	6% (-4.1)	73% (3.0)	8% (-3.9)
TH	Homogeneous			92% (0.1)	9% (-2.5)	89% (2.4)	10% (-2.1)
HY	Voxel	79% (9.6)	43% (-2.6)	52% (3.4)	31% (-1.3)	**36% (-0.9)**	3% (-5.5)
HY	Homogeneous			89% (27.0)	2% (-3.5)	107% (29.8)	2% (-4.6)
MB	Voxel	93% (4.1)	28% (-4.7)	44% (2.9)	12% (-1.7)	42% (5.2)	11% (-1.5)
MB	Homogeneous			61% (17.4)	12% (-1.5)	51% (11.9)	13% (-1.4)
P	Voxel	98% (0.0)	9% (0.0)	66% (0.0)	6% (-0.0)	61% (0.0)	6% (-0.0)
P	Homogeneous			89% (0.0)	27% (0.0)	87% (0.0)	27% (0.0)
MY	Voxel	**87% (-9.5)**	28% (-5.4)	**47% (-3.8)**	16% (-1.7)	**45% (-4.0)**	20% (-1.0)
MY	Homogeneous			59% (0.5)	3% (-0.4)	**52% (-2.2)**	2% (-0.6)
CB	Voxel	**165% (-11.5)**	1% (-8.3)	82% (3.2)	0% (-1.4)	**67% (-8.0)**	0% (-6.4)
CB	Homogeneous			101% (11.1)	1% (-3.7)	76% (12.8)	1% (-4.0)

Supplementary Table 4: Agreement in ratings across QC criteria for injection and projection QC images amongst the two raters (VN and ST). Due to its rarity, we deliberately do not show “5” failures for projections, since there was only one experiment that VN rated with a 5.

4A: Injection ratings

	**ST 0**	**ST 1**	**ST 2**	**ST 4**
**VN 0**	361	31	5	8
**VN 1**	1	3	0	0
**VN 2**	1	0	5	0
**VN 4**	2	12	2	0

4B: Projection ratings

	**ST 0**	**ST 1**	**ST 2**	**ST 3**	**ST 4**
**VN 0**	368	5	34	1	5
**VN 1**	4	0	0	0	0
**VN 2**	0	0	4	0	0
**VN 3**	0	0	0	3	0
**VN 4**	11	0	0	0	1

Supplementary Figures

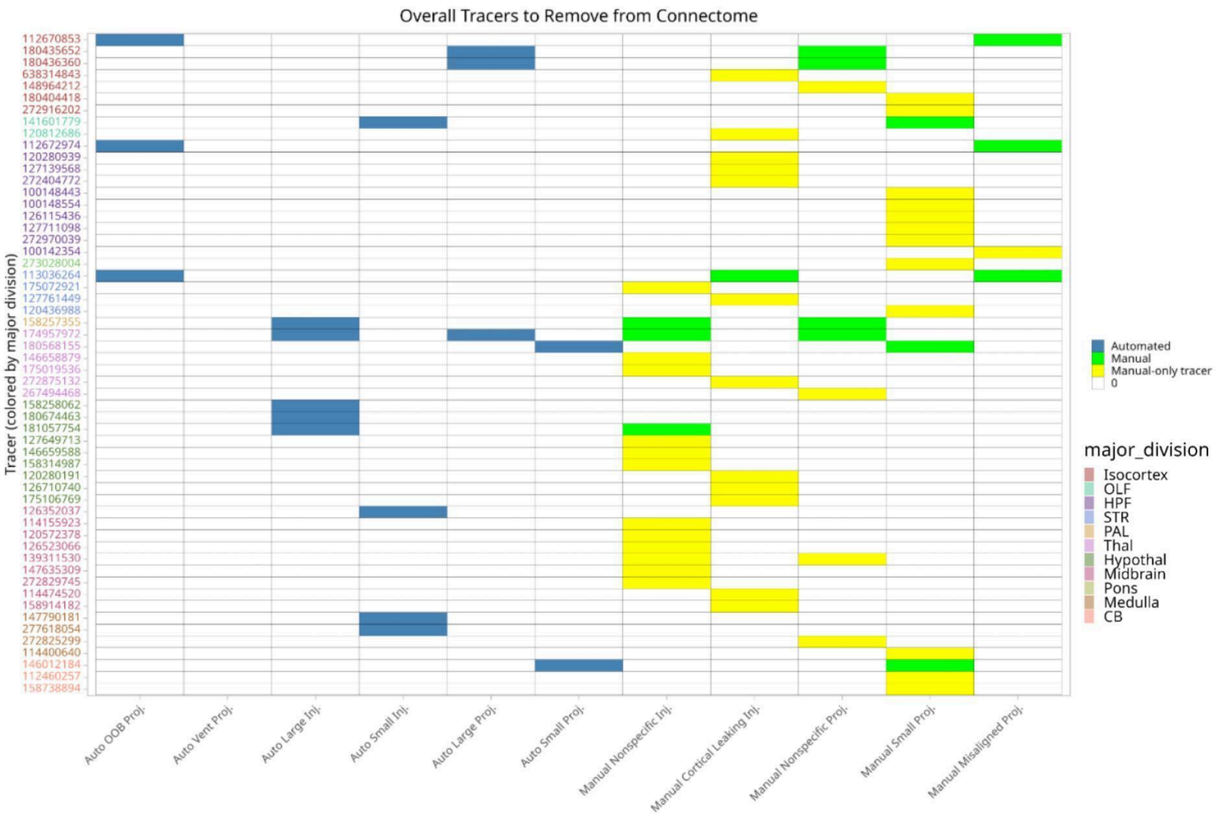

Supplementary Figure 1: The complete set of n=56 experiments removed during our QC procedure, organized by the major division of the experimental injection. For each removed experiment, we show the failure mode(s) that result in the experiment's exclusion, differentiating between automated removal criteria in blue and manual removal criteria in yellow/green. If an experiment has a green label for a manual removal criteria, then it was also flagged during automated QC.

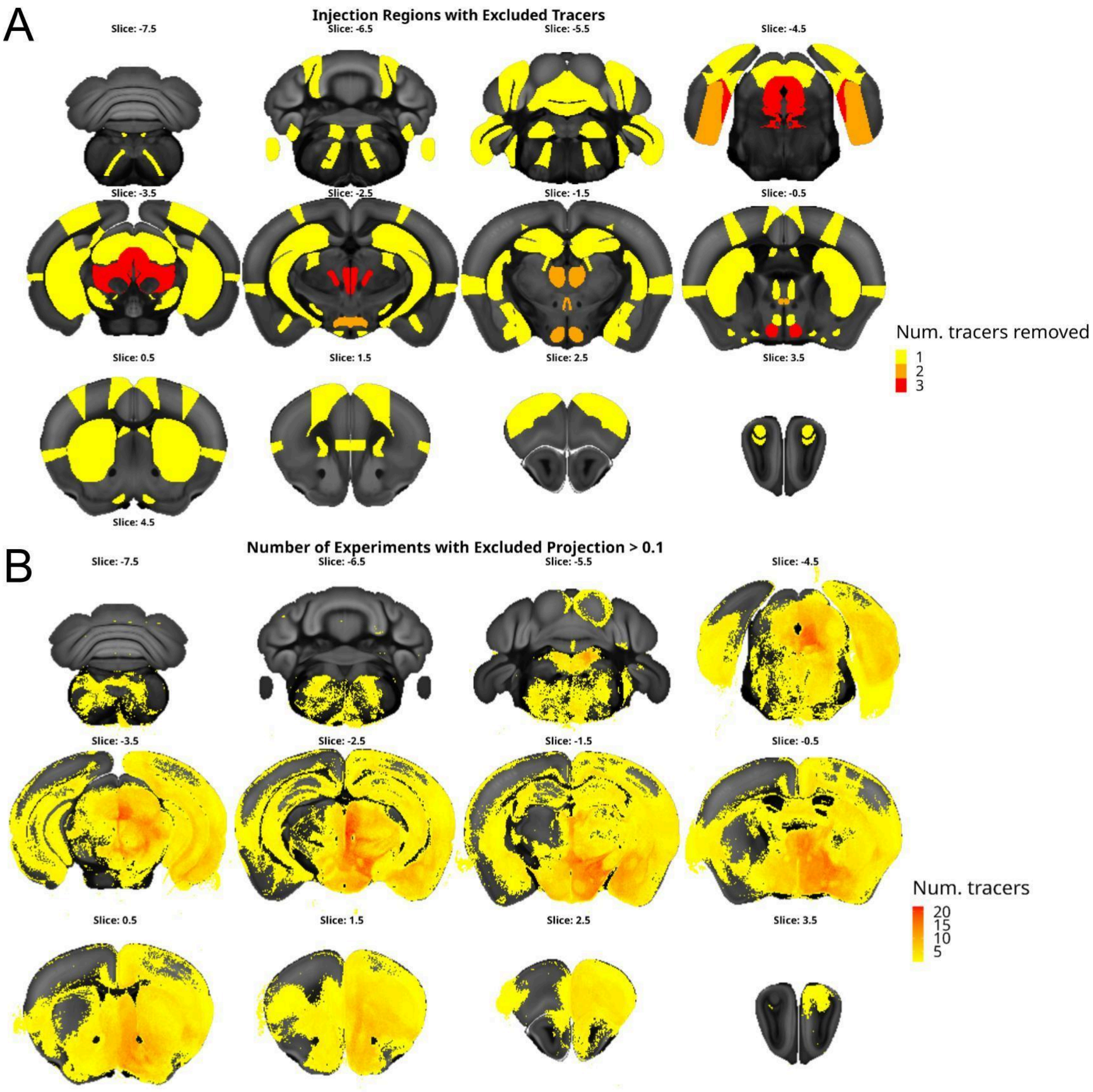
A
Injection Regions with Excluded Tracers
Slice: -7.5
Slice: -6.5
Slice: -5.5
Slice: -4.5
Slice: -3.5
Slice: -2.5
Slice: -1.5
Slice: -0.5
Slice: 0.5
Slice: 1.5
Slice: 2.5
Slice: 3.5
Slice: 4.5
Num. tracers removed
1
2
3
B
Number of Experiments with Excluded Projection > 0.1
Slice: -7.5
Slice: -6.5
Slice: -5.5
Slice: -4.5
Slice: -3.5
Slice: -2.5
Slice: -1.5
Slice: -0.5
Slice: 0.5
Slice: 1.5
Slice: 2.5
Slice: 3.5
Num. tracers
20
15
10
5

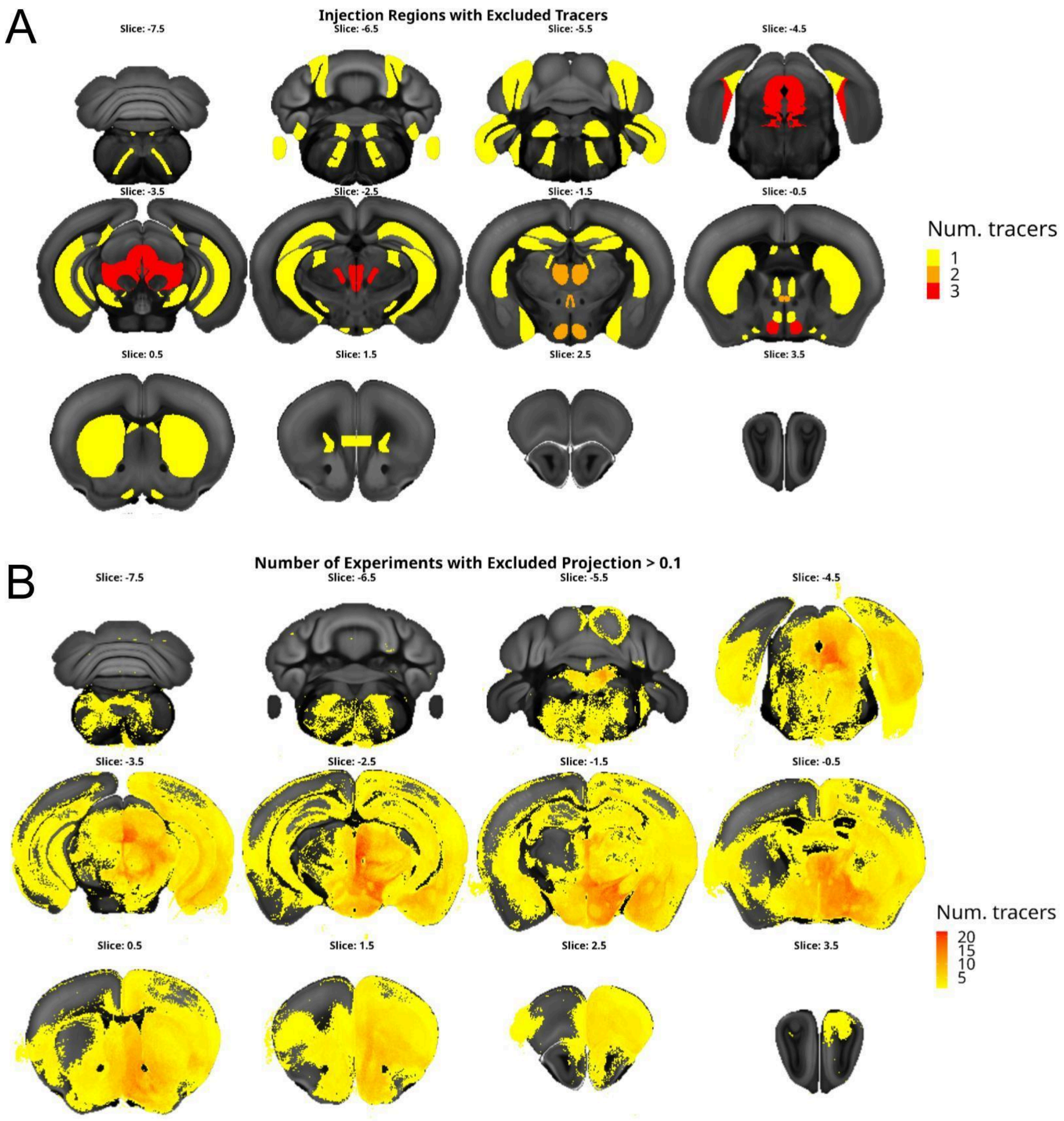

Supplementary Figure 2: (A) The number of experimental injections removed from each brain region and (B) the number of experimental projections removed from each voxel, aggregated across all n=56 removed experiments. Note that we chose to show the injections removed per region rather than per voxel due to the relative sparsity of the injection sites compared to the projection data.

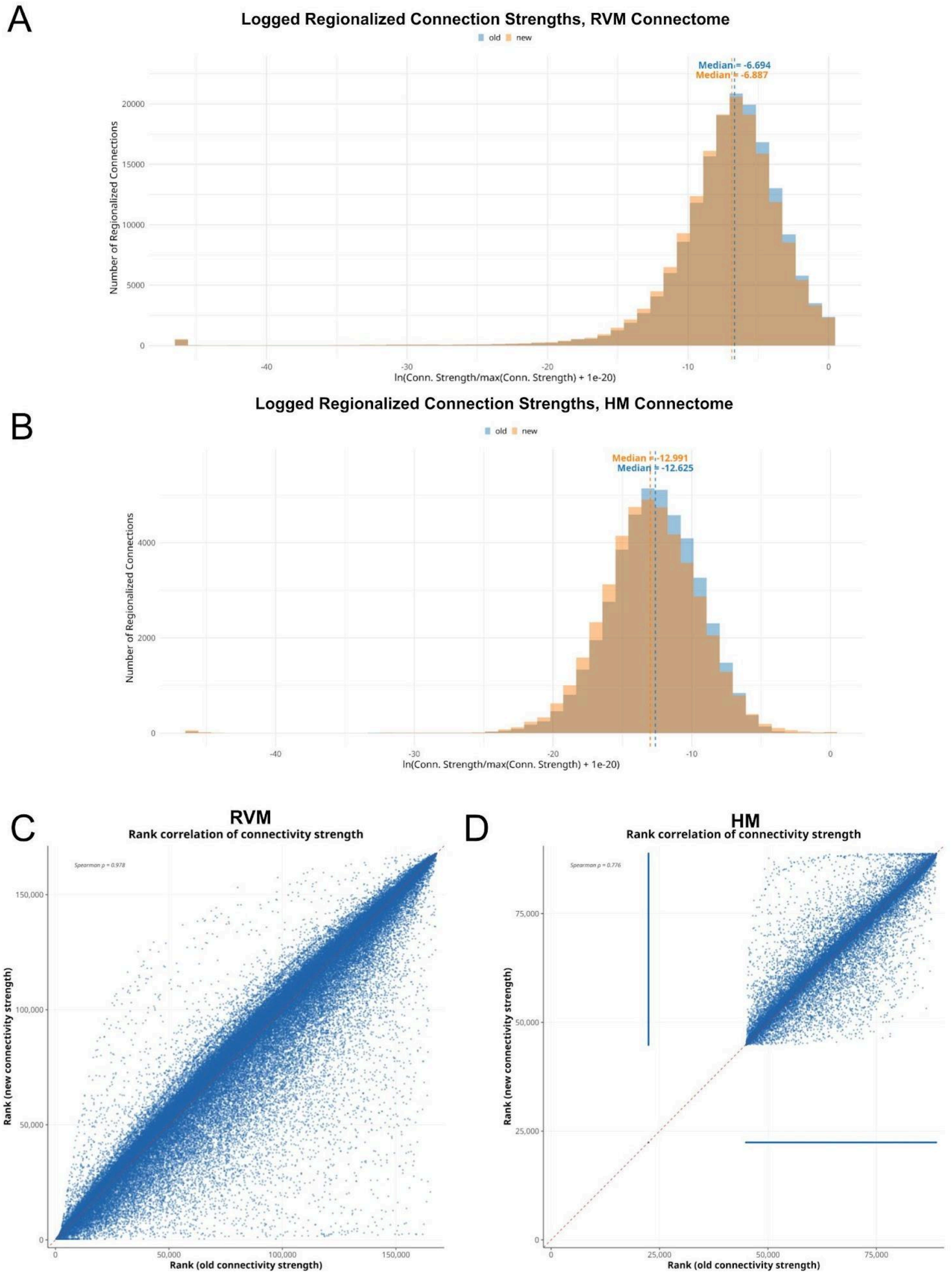

Supplementary Figure 3: Histograms showing the distributions of the logged, normalized connection strengths in (A) the RVM and (B) the HM. In each panel, we compare the distribution of the connection strengths before QC (blue) versus after QC (orange).

A

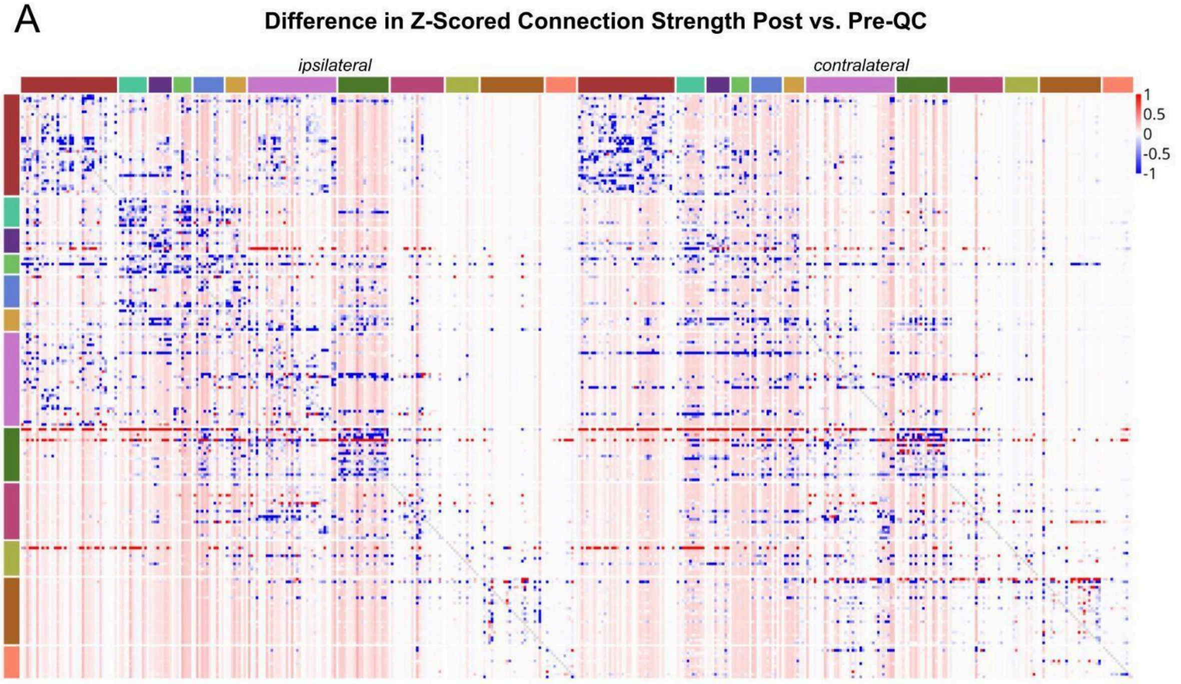

B

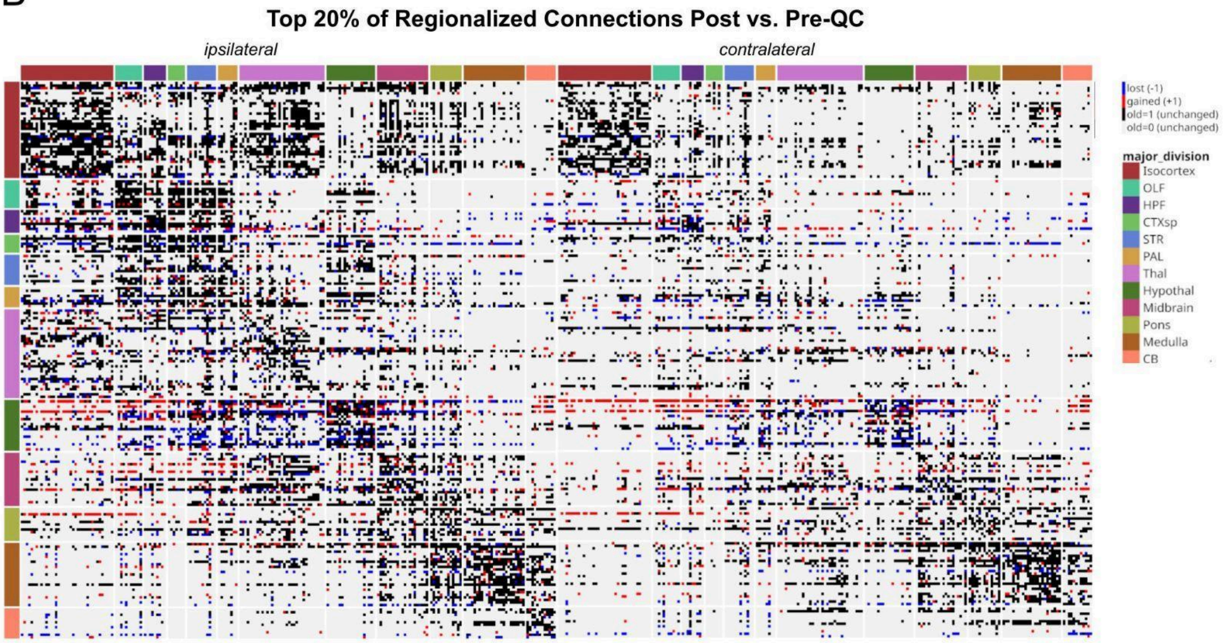

Supplementary Figure 4: Similar to Figure 4, we observe whole-brain architectural changes in the HM (n=211 regions) after QC, when examined (A) continuously, when examining the difference in z-scored, normalized connection strengths, thresholded from [-1,1] due to the presence of outliers (B) discretely, when examining the lost (blue), gained (red) and unchanged (black/gray) connections within the subset of the top 20% of the old vs. rebuilt connectomes.

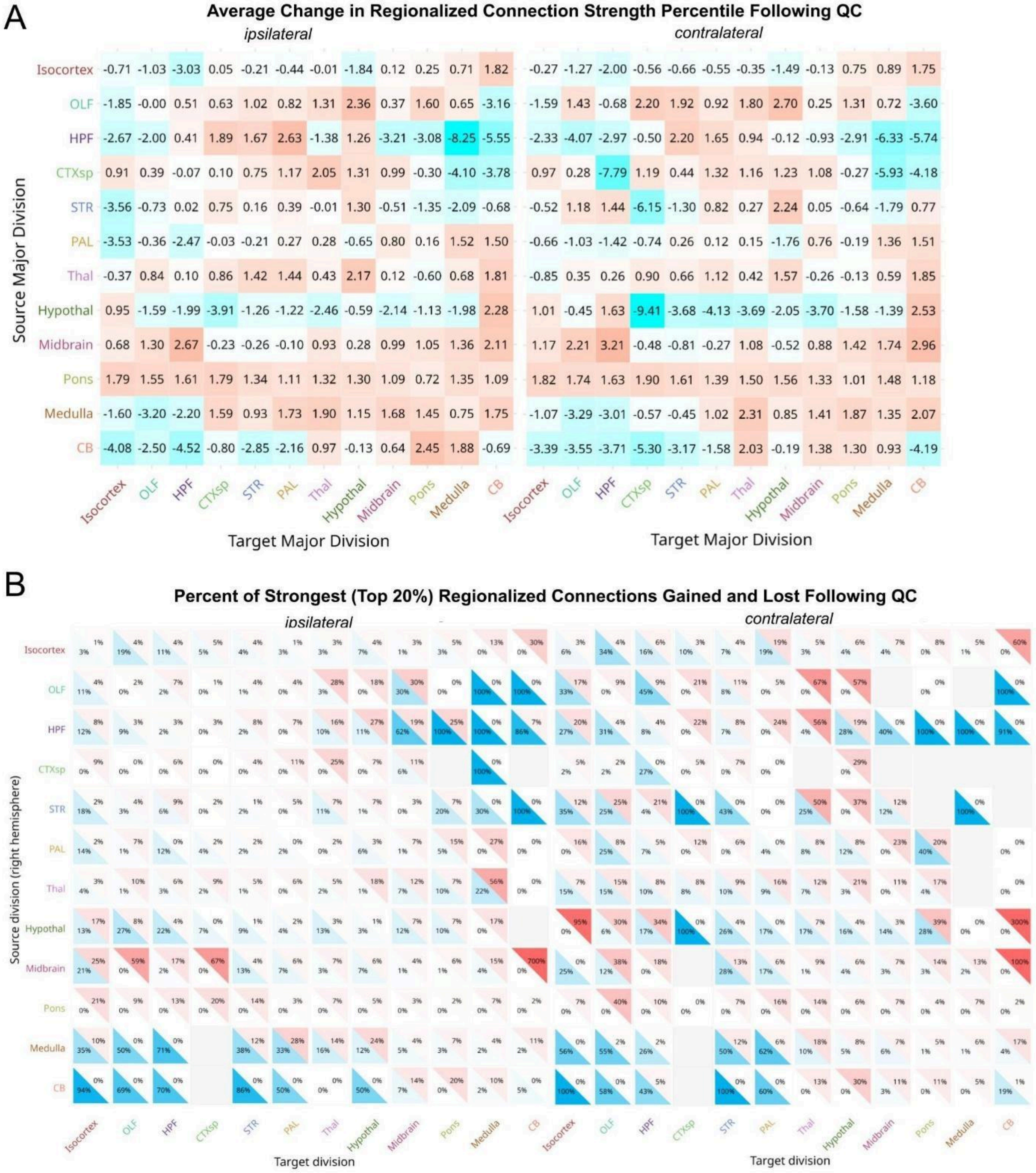

Supplementary Figure 5: We aggregate the changes in connectivity from the RVM, showing (A) the average change in connection percentile across all connections and (B) the proportion of the top 20% of connections that were lost or gained, relative to the original number of connections in each major division in the RVM, summarized across each pair of major brain divisions from Knox et al.. For both plots, blue denotes a loss in connectivity and red denotes a gain in connectivity.

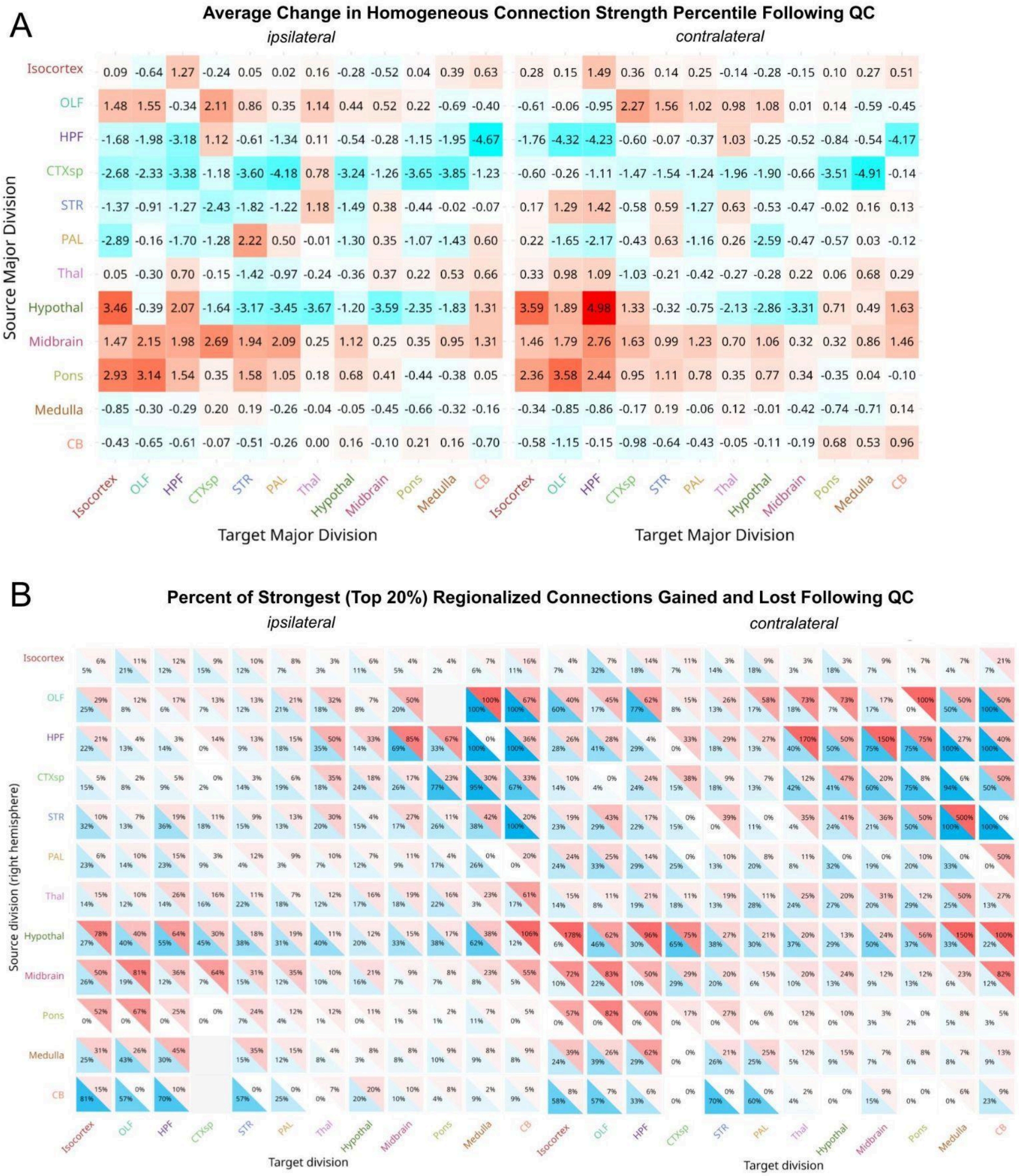

Supplementary Figure 6: Similar to Supplementary Figure 5, we aggregate the changes in connectivity from the HM, showing (A) the average change in connection percentile across all connections and (B) the proportion of the top 20% of connections that were lost or gained, summarized across each pair of major brain divisions from Oh et al.. For both plots, blue denotes a loss in connectivity and red denotes a gain in connectivity.

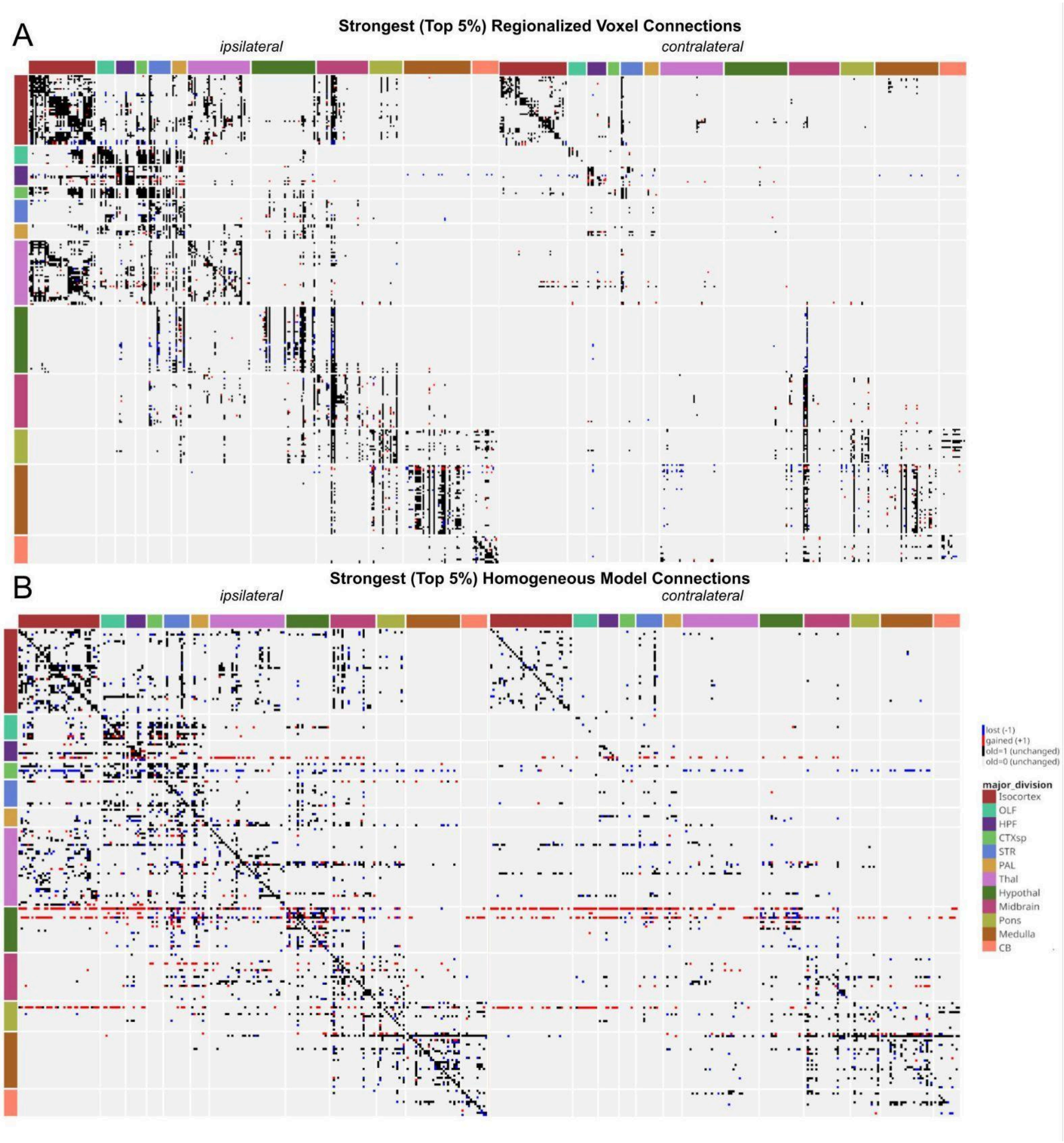

Supplementary Figure 7: Similar to Figure 4B, we observe discrete connectivity changes when examining the top 5% of connection strengths in the old vs. rebuilt connectomes. We show lost (blue), gained (red) and unchanged (black/gray) connections within the (A) RVM (B) HM.

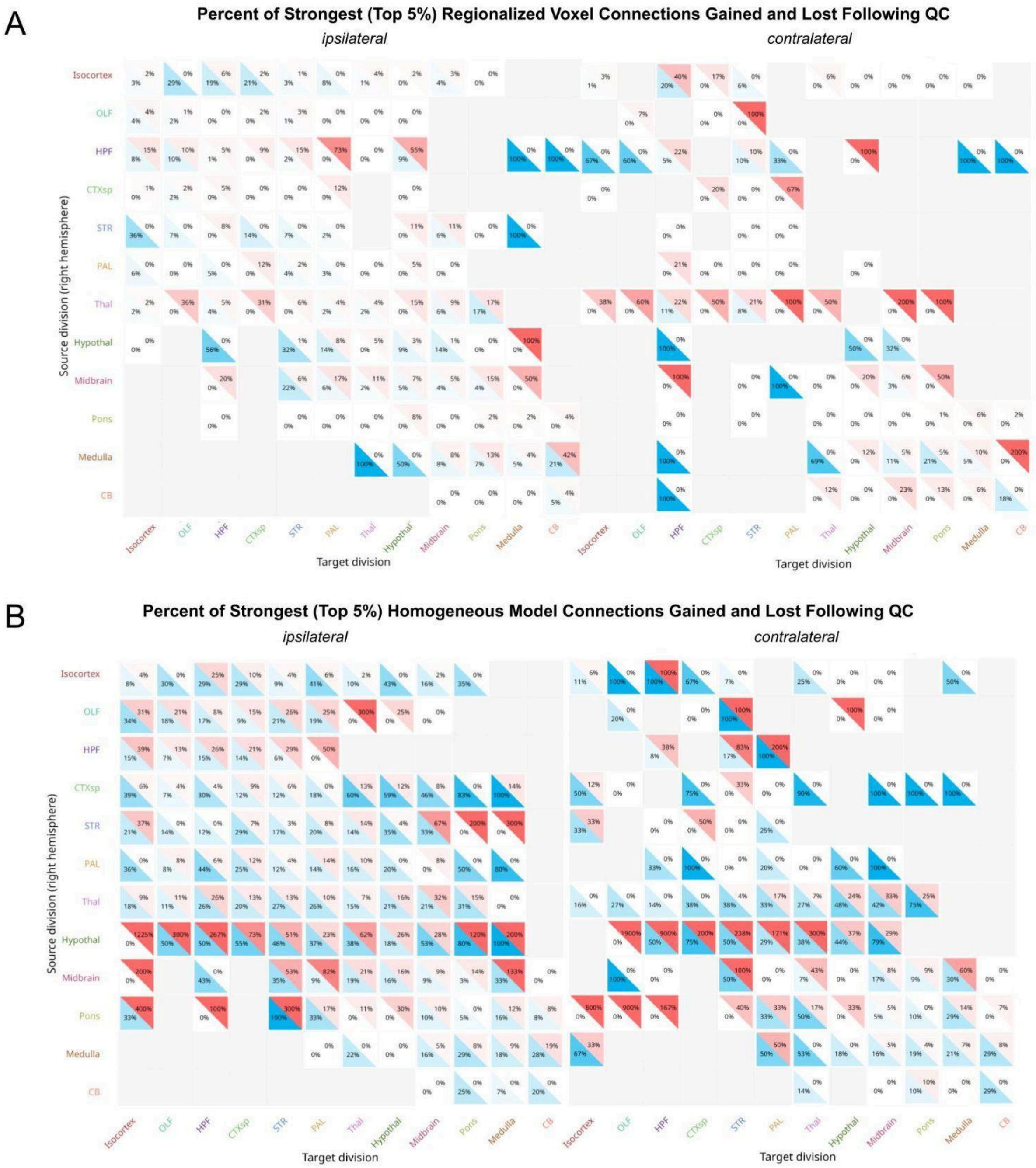

Supplementary Figure 8: Similar to Supplementary Figure 5B, we show the proportion of the top 5% of connections that were lost or gained, summarized across each pair of major brain divisions in (A) the RVM and (B) the HM.

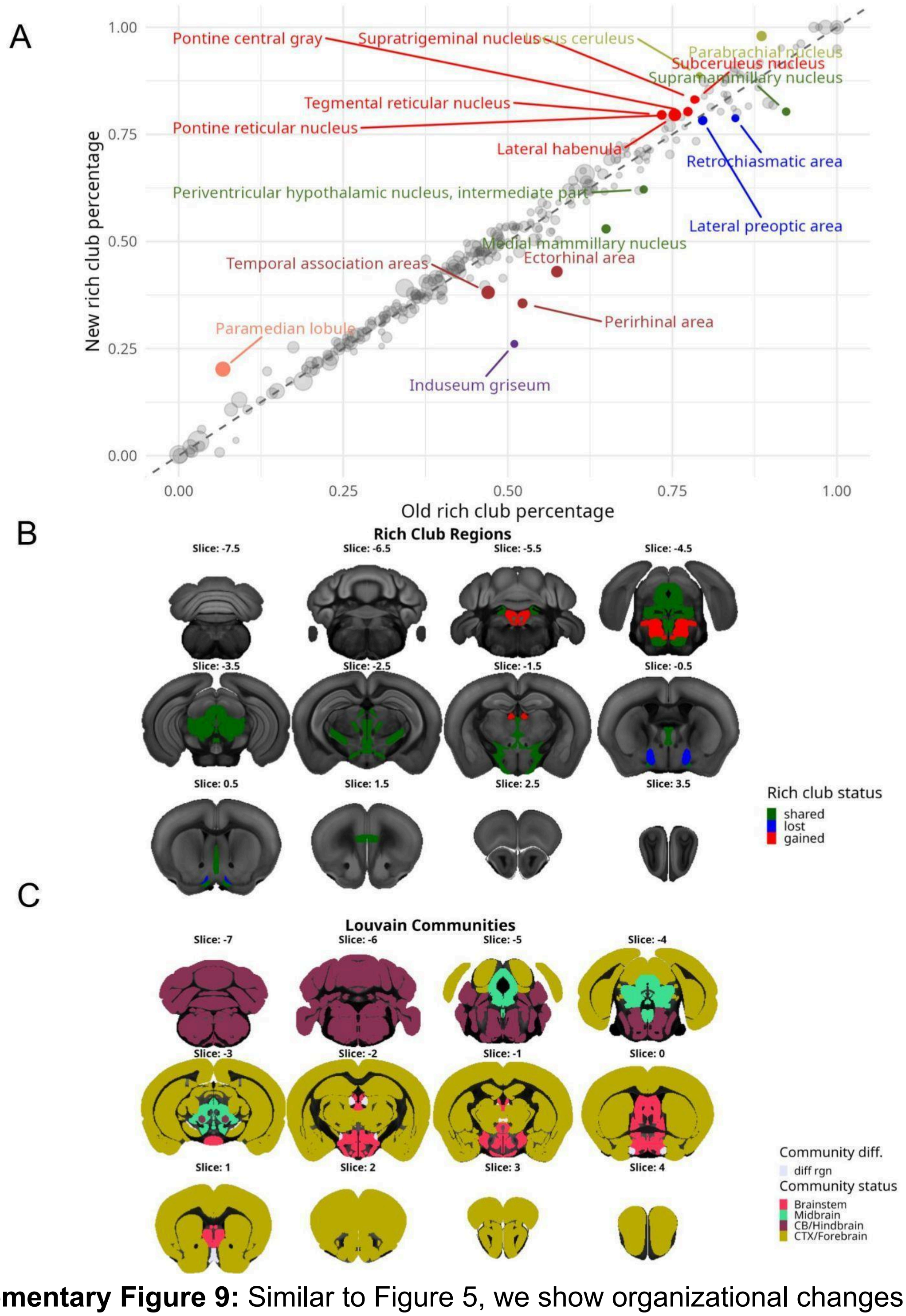

Supplementary Figure 9: Similar to Figure 5, we show organizational changes within the top 20% of connections, using the "normalized connection density" instead of the normalized connection strength in the RVM. We assume symmetric connections across hemispheres and see changes in (A) the rich club coefficient, defined as the proportion of "rich" degrees that a given region's degree is greater than or equal to (B) whether a region is "rich" or not, using a threshold of the mean plus one standard deviation of the "rich" degrees, and (C) the Louvain community assignments for each region.

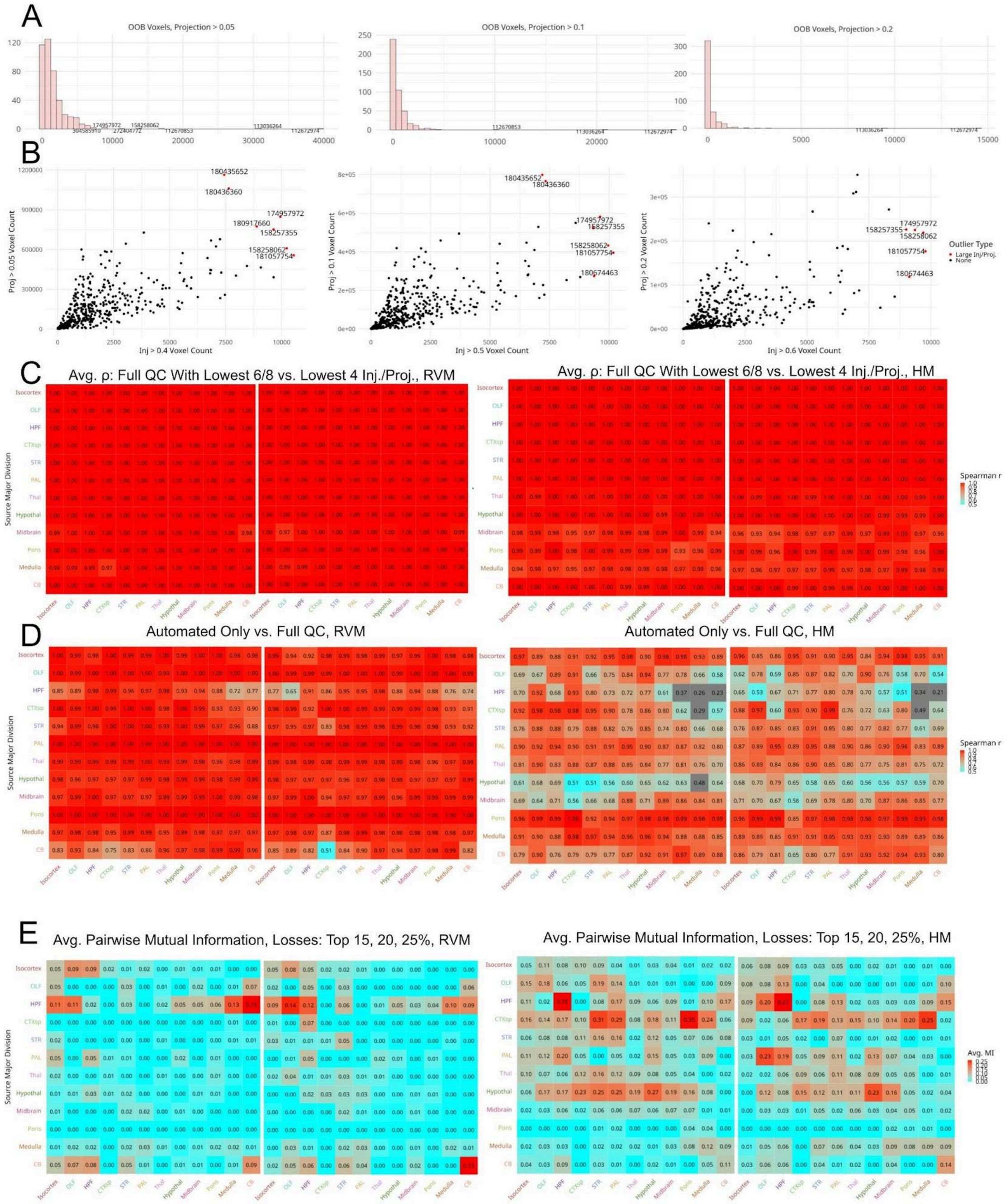

Supplementary Figure 10: Sensitivity analysis of (A) the out-of-brain outliers based on projection threshold (B) the outliers for injection and projection voxels, based on injection and projection thresholds (C) average pairwise correlation in reconstructed connection strengths within each major division, varying the number of automated lower outliers for injection/projection counts, but keeping all other excluded experiments the same (D) reconstructed connection strengths, when only excluding automated QC failures versus all manual+automated failures (E) average pairwise mutual information in the binarized “losses” in connectivity after QC, when examining the top 15, 20, and 25% of connections.

Supplementary MethodsInformation

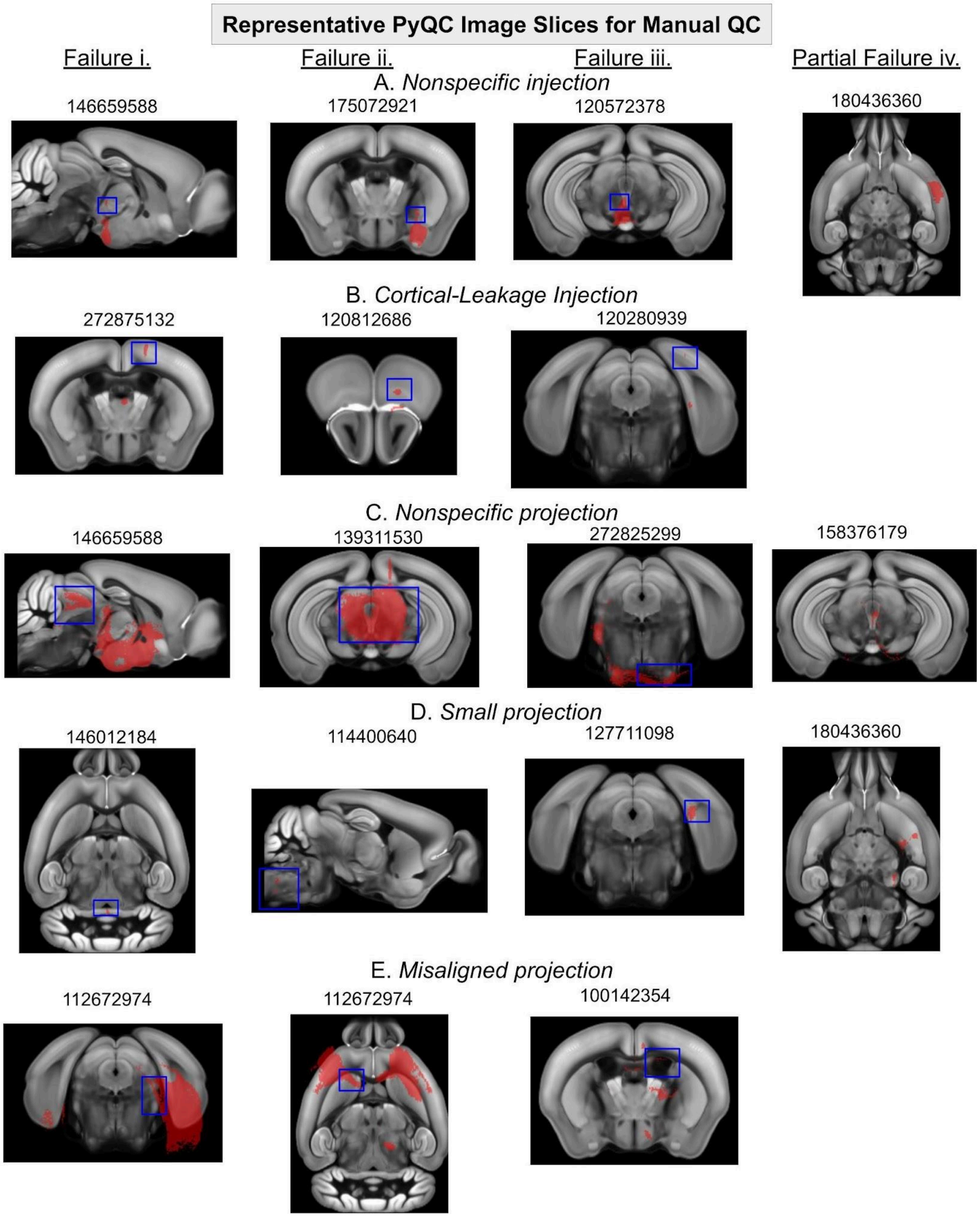

Supplementary Methods Figure 1: We show representative slice images used in PyQC for our five manual QC modalities, and highlight false-positive or false-negative connectivity patterns in blue. In (A), we show nonspecific injection volumes. We see this failure in (i) the midbrain, following a hypothalamic injection (ii) off-target striatal

injection into amygdalar-like areas (iii) nonspecific midbrain injection that infects many regions, including the highly-connected periaqueductal gray (iv) a “passed” experiment that shows a large injection, but is contained within a single cortical region. In (B), we show injection volumes with off-target “leakage” that incorrectly assign cortical connections to the (i) thalamus (ii) olfactory bulb (iii) hippocampus. In (C), we show projection volumes with “nonspecific” connectivity, which could indicate diffuse tracer spreading and “noisy” false-positive connectivity patterns (i) from the hypothalamus to the midbrain and hindbrain (ii) across a variety of midbrain regions (iii) into a number of out-of-brain voxels spanning the edge of the pons (iv) a “passed” midbrain experiment with some evidence of “noisy” spreading to the brainstem. In (D), we show projection volumes that do not spread beyond the injected region, indicating false-negative connectivity patterns to the (i) cerebellum (ii) midbrain (iii) hippocampus (iv) a “passed” striatal experiment that shows some connectivity to other regions, but is not as widespread as we would expect from the striatum. Finally, in (E), we show examples of misaligned projections, which result in the false-positive assignment of connectivity (i) from the hippocampus to the hindbrain (ii) from the corpus callosum to the striatum (iii) within the ventricles.

Extended Description: Manual QC Failures

We observe a wide variety of experiments removed across major brain divisions and failure modalities (Figure 3C), with the greatest number of removed experiments with injections in the hypothalamus, hippocampus, and midbrain. We see that manual QC is most effective at identifying cortical leaking injections (12 manual only out of 13 total removals), nonspecific injections (12/15), and small projections (12/15) from the injected region (Supplementary Figure 1). When examining the first failure mode for each experiment, we also noticed consistencies across major brain divisions. For example, all n=3 failed cerebellar experiments had small projections. Additionally, for the n=18 failed hypothalamic and midbrain experiments, all but two of the experiments had off-target injections. Nonetheless, for the hippocampal formation, we also observed a mix of connectivity failures, including out-of-brain projections (n=1), cortical leaking injections (n=3), small projections (n=5), and misaligned projections (n=1).

To examine the locations of the lost connectivity, we calculated the number of removed injections (thresholded and binarized) per Allen brain region and the number of removed projections per voxel (Supplementary Figure 2). We then divided this number by the initial counts of injections within each brain region and projections in each voxel to get the ratio of injection experiments removed per region and projection experiments removed per voxel (Figure 3B and 3C). We chose to examine injection removals per region instead of per voxel due to the relative sparsity of injection volumes compared to projection volumes, and due to the importance of injections per region in the HM from Oh et al. (2014). We find that we lose all injections into small cerebellar subregions and brainstem nuclei, which could potentially result in false-negative connectivity to these regions; however, since all other regions have at least one remaining injection, our procedure also has the potential to reduce false-positive connections (Fig. 3B) As

expected, we lose 100% of projection voxels outside of the brain and within the medial ventricle, but also lose large proportions (~75%) of projections to hypothalamic areas (Fig. 3C). Still, we see a loss in projection voxels across the brain, indicating that our QC removals are not specific to a single brain region.

Notably, among our manually excluded experiments, we observed that automated QC identified a large number of experiments with “manual nonspecific” (n=4/7) and “manual misaligned” (n=3/4) projection failures (Supplementary Figure 1). Additionally, of the n=11 experiments with both manual and automated QC failures, n=3 of these experiments failed at least three independent QC criteria (experiment IDs 158257355, 113036264, and 174957972), providing compelling evidence for removal of these experiments and the validity of our criteria. The most extreme example of this was thalamic injection experiment number 174957972, which was automatically flagged for large injection and large projection, as well as manually flagged for large injection and projection volumes.

Experimental and 2D Image Processing for Serial Two-Photon Microscopy Data
To measure connectivity to various brain regions, the Allen Institute used iontophoresis to inject a tracer into the cell bodies of various brain regions in wild-type C57BL/6J mice at P56 ± 2 postnatal days. This recombinant adeno-associated viral (rAAV) tracer expresses enhanced green fluorescent protein (EGFP) under the control of a human synapsin I promoter, thus specifically labelling neurons (Harris et al., 2012). The rAAV tracer anterogradely labels axons without crossing synapses; thus, each experiment represents directed, monosynaptic connections to the injection site. The mice were euthanized 21 days following injection before serial two-photon microscopy at a coronal slice interval of 100 μm and within-slice image resolution of 0.35 μm (Oh et al., 2014).

The scanned two-photon microscopy images were then processed by the Allen Institute. For each experiment, the 2D microscopy images first underwent intensity correction and stitching. The 2D images were then manually quality controlled (QC'd) for specimen and image quality, resulting in the removal of entire experiments with “severe artefacts” (missing tissue, low signal strength, etc.) or the masking of regions with high intensity/frequency artifacts and signal dropout (see Oh et al., 2014). Note that these QC criteria of the 2D images are completely separate from our QC criteria for the 3D images. After these corrections, the projection pixels in each image were identified by a segmentation algorithm that separated fluorescence from background noise (see Kuan et al., 2015 for more details). After segmentation, the injection site(s) were defined manually in each image by drawing closed polygons around the cell bodies of the infected neurons.

Rich Club Algorithm

1. Use *rich_club_bd.m* to calculate the rich club coefficient at each degree.
2. Generate 1000 rewired randomized networks using randmio_dir.m; calculate the rich club coefficient at each degree.

3. For each degree, see the proportion of networks from (2) that have a higher rich club coefficient than the coefficients from (1); use this to define which degrees have a significant number of nodes in a rich club
4. Identify a coefficient defining the “topological rich club” (i.e. whether a node is “rich” or not). This is equal to the mean plus one standard deviation of the degrees with significant rich clubs.
    a. Take all the nodes with degrees above this to be “rich club” nodes.
5. For each node with degree k_n, calculate the percentage of significant degrees that k_n is greater than or equal to. This is the rich club percentage, to be visualized in Fig. 5. You can now merge contralateral/ipsilateral nodes, since they will have the same rich club percentage.

Community Detection Algorithm

1. For each resolution parameter “gamma” from 0.3 to 3, in increments of 0.05:
    a. Run the Louvain community detection algorithm (community_louvain.m) 100 times.
    b. Find the node x node agreement matrix between the 100 repetitions (using consensus_und.m).
    c. Calculate the consensus community assignments from the agreement matrix by using threshold *tau*. The threshold *tau* used to binarize/cluster the agreement matrix is the mean agreement of the ***permuted*** community assignments from (a) (see Lancichinetti & Fortunato, 2012).
2. Write out the community assignments for each gamma.
3. Calculate a gamma x gamma mutual information matrix between the clusterings.
4. Determine which gamma yields the most stable clustering.
    a. For mutual information thresholds between 0.9-1 in increments of 0.005:
        i. Find the consensus clustering of the gamma x gamma matrix at the given threshold.
    b. Calculate the gamma x gamma agreement matrix across all mutual information thresholds.
    c. Visualize all values of this matrix above 0.6; heuristically select gamma in the center of the largest "cluster" of agreement matrix values. We chose gamma=1.3, as this was in the middle of a large “cluster” of agreement matrix values in the old and new regionalized voxel connectomes.
5. Run the Louvain clustering with this gamma 100 times; take the consensus clustering as the community assignments for each node.
6. Manually inspect community assignments and ensure that the numbers assigned for each community are consistent across the old versus the new connectomes. If there are two communities that have the same sets of nodes contralaterally vs. ipsilaterally, they can be merged into a single community.

Sensitivity Analysis of QC Across Parameters

Our QC, and in particular our automated QC, depends on numerous thresholds and cutoffs. To verify the robustness of our approach, we repeated our QC across a number of different parameter choices. First, for our automated QC, we varied the binarization thresholds used for the injection and projection data (Supplementary Figure 10A). We see differences in the number of experiments identified as outliers for out-of-brain voxels based on the projection threshold, ranging from n=7 to n=2 removed experiments, but note that the top two experiments for out-of-brain voxels are robustly identified across all thresholds. We also see a minimal difference in the number of upper outliers for overall injection or projection voxel counts across injection (0.4, 0.5, 0.6) and projection (0.05, 0.1, 0.2) thresholds, with n=5 out of the n=7 excluded experiments remaining the same across thresholds (Supplementary Figure 10B). We also note that reconstructed connection strengths after full QC are robust to differences in the number of automated lower outliers (6 and 8 per inj. or proj., versus the original 4), as confirmed by high average Spearman correlations of connection strengths in each major division in the RVM and HM (min $\rho=0.94$ in HM; 0.97 in RVM) (Supplementary Figure 10C).

Next, we visualized the correlation in reconstructed connectivity strengths after only removing experiments with automated QC versus all experiments (Supplementary Figure 10D). Across both the RVM (min $\rho=0.65$) and the HM (min $\rho=0.21$), the hippocampus shows the biggest difference in connectivity between the automated-only versus the full removals, indicating the importance of our manual QC in detecting the hippocampus-medulla and hippocampus-cerebellum losses in connectivity. Additionally, within the RVM, most other regions show limited differences in automated vs. full QC, whereas the HM is far more sensitive to manual QC, particularly in the hypothalamus.

Finally, we conducted a sensitivity analysis on the binarization threshold to see which binary connectivity “losses” post-QC versus pre-QC were the most consistent within the top 15, 20, and 25% of connections. (Supplementary Figure 10E). We find in the RVM that the connectivity losses with the highest average mutual information are hippocampus-medulla and hippocampus-cerebellum, indicating that these losses in connectivity are stable across binarization thresholds. However, for the HM, although the mutual information of these corresponding changes is well above zero, we see the most consistent connectivity losses in hippocampus-hippocampus connection and cortical subplate to pons connections, highlighting differences in how these models respond to QC.”

We also note that manual QC depends on the binary threshold used to generate the QC images. To verify that our manual QC failures were threshold-independent, V.N. examined all injection and projection images at a lower binarization threshold of 0.01, and did not observe notable differences in the visually-identified experimental “failures.” We include these images within the Supplementary Data.
